# Supplementary material for: Comparison of Sin-QuEChERS Nano and d-SPE Methods for Pesticide Multi-Residues in Lettuce and Chinese Chives
Source: Molecules. 2020 Jul 27;25(15):3391. doi: 10.3390/molecules25153391 (PMC7435897; doi:10.3390/molecules25153391)
Supplement: Supplementary file 1 [file molecules-25-03391-s001.pdf]

Supplementary Materials for

Comparison of Sin-QuEChERS nano and d-SPE methods for pesticide multi-residues in lettuce and Chinese chives

Yanjie Li [1], Quanshun An [2], Changpeng Zhang [1], Canping Pan [2,*] and Zhiheng Zhang [1,*]

[1] Institute of Quality and Standard for Agro-Products, Zhejiang Academy of Agricultural Sciences, Hangzhou, 310021, China; gqblyj@163.com (Y.L.); cpzhang1215@126.com (C.Z.); zhihengest@126.com (Z.Z.)

[2] Department of Applied Chemistry, College of Science, China Agricultural University, Beijing, 100193, China; anquanshun@cau.edu.cn (Q.A.); panc@cau.edu.cn (C.P.)

* Correspondence: panc@cau.edu.cn (C.P.); zhihengest@126.com (Z.Z.); Tel.: +86-10-62731978 (C.P.); +86-571- 86419053 (Z.Z.)

Table S1. Average recoveries (%) and relative standard deviation (RSDs, %) of 111 pesticides at two spiked levels (10 and 100μg/kg, n=3) in lettuce and Chinese chives.

No.	Pesticides	Lettuce, Average recovery, % (RSD, %)				Chinese chives, Average recovery, % (RSD, %)			
		Sin-QuEChERS Nano		d-SPE		Sin-QuEChERS Nano		d-SPE	
		10 μg/kg	100 μg/kg	10 μg/kg	100 μg/kg	10 μg/kg	100 μg/kg	10 μg/kg	100 μg/kg
**System: GC-MS/MS**									
1	Dichlorvos	116 (2.5)	98 (6.3)	71 (9.7)	81 (5.3)	78 (5.2)	87 (2.9)	83 (8.9)	99 (9.4)
2	Trichlorfon	118 (9.4)	108 (5.5)	80 (25)	88 (7.6)	96 (15)	90 (1.9)	92 (16)	98 (9.1)
3	Etridiazole	113 (14)	104 (10)	<LOQ	64 (11)	75 (0.1)	85 (8.9)	<LOQ	79 (2.5)
4	Carbaryl	110 (4.3)	98 (6.7)	101 (5.3)	95 (3.2)	116 (5.3)	103 (2.2)	117 (5.1)	106 (2.2)
5	Orthophenylphenol	98 (9.1)	102 (7.3)	98 (5.9)	102 (7.7)	99 (2.2)	99 (0.9)	119 (4.4)	108 (1.4)
6	Propachlor	110 (9.0)	99 (0.9)	80 (18)	93 (4.0)	111 (1.3)	99 (9.8)	84 (6.0)	95 (5.5)
7	Ethoprophos	100 (6.4)	101 (2.0)	71 (14)	89 (3.8)	91 (13)	106 (2.3)	100 (3.7)	102 (1.8)
8	Chlorpropham	87 (11)	102 (6.9)	82 (12)	101 (6.2)	97 (1.9)	103 (1.6)	97 (4.5)	106 (2.5)
9	Trifluralin	92 (9.2)	101 (3.6)	89 (7.2)	93 (2.4)	104 (8.4)	98 (2.2)	93 (2.3)	100 (1.6)
10	Sulfotep	89 (7.9)	99 (3.7)	84 (11)	99 (2.9)	101 (6.4)	106 (2.0)	88 (5.5)	104 (5.4)
11	Phorate	84 (12)	97 (1.6)	68 (10)	89 (1.6)	101 (12)	101 (4.1)	99 (6.1)	107 (2.7)
12	Atrazine	101 (13)	91 (3.6)	108 (3.0)	99 (4.4)	93 (15)	94 (1.3)	90 (9.8)	107 (3.9)

No.	Pesticides	Lettuce, Average recovery, % (RSD, %)				Chinese chives, Average recovery, % (RSD, %)			
		Sin-QuEChERS Nano		d-SPE		Sin-QuEChERS Nano		d-SPE	
		10 μg/kg	100 μg/kg	10 μg/kg	100 μg/kg	10 μg/kg	100 μg/kg	10 μg/kg	100 μg/kg
13	Propazine	94 (13)	102 (4.3)	83 (4.9)	105 (1.2)	92 (9.4)	100 (1.6)	97 (10)	106 (5.4)
14	Clomazone	99 (4.4)	97 (5.3)	82 (14)	88 (5.3)	96 (11)	104 (2.0)	102 (11)	104 (2.8)
15	Lindane	85 (15)	105 (7.8)	75 (13)	86 (13)	87 (13)	97 (0.8)	80 (22)	95 (4.5)
16	Propyzamide	79 (13)	94 (2.9)	89 (6.6)	92 (1.8)	89 (14)	99 (8.4)	107 (1.6)	99 (6.6)
17	Diazinon	95 (2.1)	101 (3.1)	89 (9.0)	101 (2.4)	97 (4.8)	98 (4.4)	104 (10)	107 (0.4)
18	Triallate	92 (11)	102 (1.7)	85 (9.5)	97 (3.2)	95 (5.1)	97 (1.9)	92 (10)	103 (4.1)
19	Pirimicarb	92 (2.9)	94 (5.1)	79 (13)	91 (2.3)	82 (1.7)	97 (1.7)	102 (4.8)	110 (4.5)
20	Propanil	80 (34)	85 (4.4)	80 (12)	83 (3.8)	86 (6.1)	94 (5.9)	91 (9.8)	99 (5.6)
21	Acetochlor	85 (25)	96 (3.8)	91 (7.7)	90 (8.2)	119 (5.0)	96 (6.4)	82 (5.9)	102 (8.6)
22	Propisochlor	103 (4.8)	95 (4.4)	88 (13)	100 (10)	97 (13)	92 (2.1)	87 (11)	95 (8.3)
23	Metribuzin	82 (15)	98 (4.9)	78 (9.6)	98 (3.5)	79 (9.1)	98 (2.0)	94 (5.4)	105 (1.8)
24	Vinclozolin	87 (12)	94 (3.0)	80 (6.6)	96 (3.2)	83 (9.1)	95 (4.5)	84 (30)	104 (5.9)
25	Parathion-Methyl	85 (8.3)	100 (0.7)	70 (9.6)	74 (8.0)	104 (8.1)	102 (3.6)	84 (6.9)	103 (5.1)
26	Tolclofos-Methyl	105 (4.3)	104 (6.3)	96 (12)	95 (1.7)	97 (6.1)	99 (2.3)	102 (6.3)	109 (1.1)
27	Metalaxyl	92 (3.3)	96 (3.1)	102 (6.9)	106 (5.1)	113 (2.4)	106 (11)	110 (8.2)	101 (4.3)
28	Ametryn	85 (2.4)	97 (4.0)	92 (4.7)	99 (2.0)	86 (6.8)	93 (3.2)	101 (0.8)	108 (3.3)
29	Prometryn	104 (8.2)	100 (1.6)	91 (4.8)	106 (2.9)	93 (1.7)	95 (1.9)	98 (9.9)	106 (5.8)
30	Pirimiphos-Methyl	85 (9.7)	94 (5.8)	88 (1.2)	87 (4.8)	108 (8.7)	94 (4.4)	96 (11)	99 (10)
31	Fenitrothion	90 (8.9)	79 (12)	76 (12)	82 (10)	81 (11)	85 (9.2)	70 (22)	90 (6.4)
32	Malathion	108 (1.4)	99 (6.9)	78 (15)	84 (5.5)	117 (6.5)	103 (3.3)	110 (11)	106 (3.3)
33	Metolachlor	98 (4.5)	102 (3.9)	91 (7.8)	102 (3.1)	99 (4.1)	100 (1.9)	101 (2.9)	108 (2.4)
34	Diethofencarb	101 (9.9)	106 (4.0)	78 (13)	98 (5.8)	94 (6.8)	93 (4.9)	103 (4.3)	101 (6.3)
35	Triadimefon	85 (4.8)	94 (3.7)	84 (8.1)	104 (4.2)	115 (11)	96 (2.7)	85 (15)	100 (3.7)
36	Thiametoxam	88 (14)	95 (12)	70 (5.6)	79 (2.1)	85 (4.7)	99 (2.2)	100 (6.5)	96 (7.9)
37	Pendimethalin	82 (9.9)	88 (3.9)	71 (8.0)	93 (4.7)	106 (6.0)	86 (5.2)	82 (0.9)	108 (7.2)

No.	Pesticides	Lettuce, Average recovery, % (RSD, %)				Chinese chives, Average recovery, % (RSD, %)			
		Sin-QuEChERS Nano		d-SPE		Sin-QuEChERS Nano		d-SPE	
		10 µg/kg	100 µg/kg	10 µg/kg	100 µg/kg	10 µg/kg	100 µg/kg	10 µg/kg	100 µg/kg
38	Fipronil	87 (5.4)	98 (3.7)	81 (7.7)	95 (6.5)	86 (7.2)	96 (5.5)	89 (4.0)	86 (5.6)
39	Penconazole	97 (8.7)	104 (5.4)	87 (8.9)	102 (0.4)	97 (2.5)	96 (1.7)	101 (8.2)	103 (5.0)
40	Chlorfenvinphos	92 (12.4)	104 (6.7)	78 (8.4)	90 (3.7)	101 (1.7)	102 (3.7)	95 (5.6)	105 (2.7)
41	Phenthoate	87 (11.9)	100 (2.6)	82 (6.0)	93 (2.5)	86 (7.7)	96 (1.6)	83 (3.0)	102 (2.4)
42	Triadimenol	99 (6.0)	105 (0.8)	92 (11)	102 (3.1)	104 (1.4)	98 (2.1)	104 (3.6)	97 (2.6)
43	Methidathion	80 (9.3)	91 (14)	70 (20)	78 (3.5)	92 (8.5)	97 (2.1)	79 (20)	93 (2.2)
44	Butachlor	94 (3.1)	103 (8.2)	91 (17)	101 (3.6)	89 (2.3)	100 (8.2)	120 (20)	105 (1.5)
45	Napropamide	94 (11)	105 (3.1)	88 (9.3)	99 (5.0)	81 (12)	90 (5.5)	109 (5.7)	105 (2.9)
46	Pretilachlor	104 (13)	109 (4.4)	90 (1.9)	102 (3.5)	98 (13)	97 (4.2)	105 (3.4)	105 (2.8)
47	Isoprothiolane	79 (8.0)	100 (1.4)	77 (1.2)	111 (1.8)	102 (12)	94 (8.9)	83 (23)	101 (1.5)
48	Oxadiazon	108 (20)	104 (5.9)	92 (6.0)	100 (2.6)	104 (6.9)	96 (4.0)	107 (14)	109 (1.6)
49	Thifluzamide	107 (2.2)	111 (4.8)	82 (6.3)	101 (1.5)	96 (8.8)	102 (3.0)	104 (6.4)	105 (4.4)
50	Myclobutanil	104 (11)	101 (4.9)	86 (2.4)	102 (1.7)	97 (6.3)	103 (1.0)	102 (6.5)	107 (3.1)
51	o,p'-DDT	83 (6.9)	106 (5.7)	93 (22)	102 (6.2)	88 (9.3)	101 (1.7)	97 (7.0)	98 (5.0)
52	Kresoxim-Methyl	93 (9.0)	105 (2.7)	88 (3.9)	101 (3.6)	99 (4.2)	98 (1.9)	94 (8.2)	106 (2.9)
53	Phosmet	105 (8.9)	107 (5.5)	90 (4.7)	106 (2.3)	111 (7.4)	104 (1.4)	109 (7.3)	111 (4.7)
54	Trifloxystrobin	101 (15)	105 (4.3)	97 (5.6)	101 (5.3)	107 (6.8)	104 (3.7)	115 (8.6)	111 (4.1)
55	Cyproconazole	95 (8.7)	101 (5.4)	88 (1.8)	90 (1.0)	91 (9.4)	100 (4.5)	96 (13)	100 (1.5)
56	Diniconazole	98 (2.1)	96 (3.6)	81 (5.1)	98 (4.3)	77 (15)	81 (11)	80 (3.5)	108 (1.8)
57	Oxadixyl	96 (2.0)	95 (3.2)	79 (4.8)	100 (2.9)	106 (8.5)	97 (2.3)	99 (8.7)	104 (3.7)
58	Carfentrazone-ethyl	91 (11)	97 (0.4)	92 (7.9)	96 (4.0)	118 (3.5)	94 (2.6)	111 (11)	106 (13)
59	Clodinafop-propargyl	75 (2.7)	99 (9.7)	74 (4.6)	83 (4.5)	96 (1.0)	95 (0.4)	97 (2.2)	104 (2.0)
60	Tebuconazole	90 (13)	101 (2.1)	76 (8.7)	92 (5.6)	88 (9.9)	94 (5.4)	98 (5.5)	102 (2.3)
61	Diclofopmethyl	101 (6.1)	103 (7.1)	76 (9.6)	96 (2.9)	82 (3.4)	96 (0.4)	99 (9.0)	104 (4.1)
62	Propargite	81 (15)	99 (9.3)	88 (2.1)	91 (6.1)	89 (2.9)	99 (2.8)	84 (12)	103 (4.0)

No.	Pesticides	Lettuce, Average recovery, % (RSD, %)				Chinese chives, Average recovery, % (RSD, %)			
		Sin-QuEChERS Nano		d-SPE		Sin-QuEChERS Nano		d-SPE	
		10 μg/kg	100 μg/kg	10 μg/kg	100 μg/kg	10 μg/kg	100 μg/kg	10 μg/kg	100 μg/kg
63	Bifenthrin	95 (11)	101 (2.1)	99 (7.4)	104 (2.6)	97 (9.1)	95 (2.7)	109 (6.4)	106 (2.3)
64	Cypermethrin	99 (4.8)	103 (8.4)	83 (30)	97 (4.8)	83 (11)	93 (1.0)	90 (24)	102 (4.1)
65	Fenpropathrin	102 (7.2)	96 (4.9)	76 (10)	83 (3.0)	109 (9.4)	98 (5.7)	100 (13)	101 (7.2)
66	Lambda-Cyhalothrin	90 (4.3)	101 (5.4)	79 (6.0)	90 (2.1)	95 (4.5)	99 (0.8)	97 (7.3)	102 (5.3)
67	Triticonazole	87 (7.9)	99 (3.7)	77 (2.0)	92 (2.4)	110 (9.1)	93 (12)	94 (8.2)	100 (2.4)
68	Pyriproxyfen	83 (12)	99 (3.8)	82 (5.1)	101 (2.4)	84 (11)	92 (3.4)	103 (7.6)	107 (2.7)
69	Cyhalofopbutyl	90 (9.9)	101 (5.0)	85 (4.7)	98 (1.3)	94 (7.2)	95 (2.0)	100 (0.6)	106 (1.9)
70	Permethrin	91 (8.9)	101 (4.2)	91 (8.7)	99 (4.5)	85 (9.1)	93 (5.2)	116 (1.4)	108 (2.4)
71	Pyridaben	99 (7.1)	106 (5.7)	84 (6.3)	98 (2.3)	95 (7.4)	99 (3.5)	101 (3.4)	106 (3.4)
72	Beta-cypermethrin	104 (9.8)	106 (5.8)	70 (10)	80 (3.4)	103 (7.9)	96 (2.3)	111 (5.4)	104 (3.0)
73	Flumioxazin	107 (13)	113 (1.6)	71 (14)	87 (4.9)	88 (3.5)	107 (5.8)	100 (12)	94 (5.1)
74	Esfenvalerate	109 (8.1)	111 (12)	76 (9.2)	78 (3.4)	86 (13)	98 (6.4)	90 (2.5)	101 (4.0)
75	Famoxadone	122 (14)	111 (9.4)	73 (15)	72 (3.3)	88 (7.6)	104 (2.5)	84 (14)	96 (6.6)
**System: LC-MS/MS**									
1	Omethoate	136 (2.7)	124 (7.5)	96 (0.7)	100 (2.1)	101 (10)	91 (1.3)	78 (9.8)	88 (7.7)
2	Methomyl	100 (9.8)	96 (4.7)	103 (15)	114 (0.7)	95 (12)	91 (0.6)	132 (3.4)	100 (2.9)
3	Thiamethoxam	105 (9.8)	101 (0.7)	114 (8.7)	101 (3.5)	106 (3.4)	96 (2.7)	106 (10)	96 (3.6)
4	Metamitron	83 (5.7)	88 (2.0)	113 (1.0)	105 (2.0)	88 (11)	93 (6.2)	91 (5.9)	97 (3.0)
5	Clothianidin	109 (8.1)	105 (3.7)	103 (8.4)	101 (2.9)	92 (7.0)	99 (2.0)	93 (7.0)	93 (3.1)
6	Imidacloprid	108 (6.1)	97 (1.6)	118 (8.7)	101 (0.9)	98 (6.5)	105 (0.8)	87 (7.5)	95 (2.8)
7	Dimethoate	104 (6.4)	99 (3.3)	95 (1.5)	102 (1.7)	105 (2.8)	101 (2.6)	87 (5.7)	99 (3.4)
8	Acetamiprid	106 (2.2)	96 (1.2)	94 (6.3)	103 (2.7)	104 (10)	102 (2.5)	89 (9.6)	95 (2.0)
9	Cymoxanil	111 (11)	97 (3.4)	99 (11)	103 (4.2)	83 (10)	84 (6.8)	81 (15)	77 (3.3)
10	Thiacloprid	105 (4.0)	100 (1.8)	104 (5.9)	104 (5.9)	100 (4.3)	95 (1.8)	82 (5.4)	89 (2.2)
11	Imazalil	92 (3.8)	97 (0.3)	72 (10)	82 (5.0)	87 (4.0)	85 (4.0)	77 (9.3)	83 (2.4)

No.	Pesticides	Lettuce, Average recovery, % (RSD, %)				Chinese chives, Average recovery, % (RSD, %)			
		Sin-QuEChERS Nano		d-SPE		Sin-QuEChERS Nano		d-SPE	
		10 μg/kg	100 μg/kg	10 μg/kg	100 μg/kg	10 μg/kg	100 μg/kg	10 μg/kg	100 μg/kg
12	Metribuzin	105 (4.8)	104 (0.4)	117 (5.0)	107 (1.0)	97 (4.1)	104 (2.3)	104 (4.3)	98 (3.2)
13	Bendiocarb	99 (2.9)	95 (2.6)	99 (2.4)	102 (3.6)	102 (3.6)	95 (2.8)	107 (8.7)	101 (0.5)
14	Carbofuran	106 (1.2)	108 (1.4)	118 (3.4)	111 (0.3)	106 (1.3)	104 (1.7)	99 (4.8)	105 (2.6)
15	Carbaryl	96 (0.5)	100 (4.8)	92 (13)	99 (3.1)	103 (7.2)	104 (2.6)	102 (11)	98 (2.9)
16	Isoproturon	98 (3.5)	97 (1.1)	114 (3.9)	110 (3.1)	105 (5.5)	100 (1.6)	95 (5.9)	106 (4.3)
17	Metalaxyl	98 (3.9)	100 (1.3)	113 (2.8)	110 (1.1)	101 (0.3)	102 (2.9)	111 (3.7)	97 (1.8)
18	Propanil	100 (9.6)	94 (0.9)	96 (2.4)	104 (4.1)	78 (11)	91 (5.3)	84 (14)	89 (1.6)
19	Dimethomorph	94 (12)	105 (4.6)	113 (6.1)	109 (3.7)	106 (8.9)	91 (4.9)	101 (6.9)	107 (1.8)
20	Cyprodinil	41 (2.3)	45 (2.1)	103 (7.1)	99 (2.8)	25 (42)	28 (42)	87 (1.1)	93 (2.3)
21	Triadimenol	91 (3.1)	101 (1.2)	111 (8.0)	103 (2.2)	93 (3.9)	102 (2.9)	97 (4.5)	98 (4.0)
22	Cyproconazole	101 (9.7)	102 (2.6)	108 (10)	107 (3.0)	103 (13)	103 (3.0)	89 (2.8)	100 (2.3)
23	Fludioxonil	87 (12)	92 (4.0)	95 (4.1)	100 (1.4)	112 (11)	101 (14)	106 (12)	101 (11)
24	Triticonazole	113 (0.8)	110 (3.6)	95 (5.0)	101 (0.4)	102 (5.1)	102 (1.2)	105 (1.6)	101 (4.1)
25	Diethofencarb	85 (5.3)	101 (7.1)	86 (15)	99 (4.0)	95 (8.8)	95 (4.2)	108 (4.8)	96 (6.1)
26	Spirotetramat	129 (9.5)	100 (2.7)	83 (9.6)	105 (5.9)	110 (5.3)	112 (4.8)	94 (3.4)	82 (7.0)
27	Prochloraz	92 (2.3)	88 (5.5)	70 (3.7)	95 (6.4)	88 (4.9)	87 (2.7)	96 (3.1)	98 (2.5)
28	Myclobutanil	96 (2.0)	112 (3.3)	91 (7.8)	103 (3.0)	98 (7.4)	101 (2.1)	108 (6.1)	103 (2.4)
29	Azoxystrobin	97 (4.2)	105 (2.4)	82 (12)	104 (6.4)	107 (12)	106 (2.0)	89 (6.5)	104 (0.8)
30	Triadimefon	114 (0.8)	101 (0.9)	106 (5.3)	110 (6.8)	78 (9.4)	106 (1.6)	76 (6.5)	99 (1.3)
31	Epoxiconazole	102 (4.3)	101 (2.0)	108 (2.2)	96 (6.3)	94 (4.2)	100 (2.7)	86 (10)	102 (5.1)
32	Ethoprophos	93 (5.5)	95 (2.2)	108 (4.8)	107 (6.1)	94 (14)	102 (3.1)	89 (5.2)	95 (6.0)
33	Fluopyram	94 (8.9)	102 (1.8)	101 (6.3)	99 (4.9)	93 (5.6)	100 (0.9)	91 (0.2)	102 (2.2)
34	Tebuconazole	103 (4.3)	99 (2.3)	97 (2.4)	96 (6.4)	87 (6.6)	99 (3.7)	91 (2.3)	97 (2.5)
35	Hexaconazole	104 (4.7)	101 (1.9)	97 (6.6)	99 (8.4)	101 (4.7)	102 (2.8)	89 (3.6)	100 (1.5)
36	Penconazole	101 (3.1)	97 (1.6)	103 (3.4)	98 (7.4)	104 (4.7)	98 (0.3)	102 (3.2)	100 (1.9)

No.	Pesticides	Lettuce, Average recovery, % (RSD, %)				Chinese chives, Average recovery, % (RSD, %)			
		Sin-QuEChERS Nano		d-SPE		Sin-QuEChERS Nano		d-SPE	
		10 μg/kg	100 μg/kg	10 μg/kg	100 μg/kg	10 μg/kg	100 μg/kg	10 μg/kg	100 μg/kg
37	Triazophos	104 (3.0)	101 (0.4)	92 (1.1)	102 (4.2)	95 (1.8)	101 (4.5)	89 (11)	101 (3.1)
38	Metolachlor	98 (5.0)	98 (3.0)	97 (1.8)	102 (5.6)	98 (9.9)	104 (2.3)	88 (5.5)	95 (1.5)
39	Diniconazole	105 (9.1)	98 (1.0)	98 (2.2)	94 (12)	77 (9.8)	88 (8.8)	78 (9.9)	97 (2.3)
40	Propiconazole	99 (7.2)	100 (0.6)	100 (4.5)	105 (6.9)	98 (6.3)	97 (1.7)	92 (9.0)	99 (0.9)
41	Tebufenozide	100 (1.3)	105 (3.9)	96 (2.3)	99 (6.8)	98 (3.5)	101 (1.2)	99 (4.1)	96 (5.4)
42	Chlorfenvinphos	112 (1.3)	108 (4.6)	93 (13)	103 (9.1)	114 (9.9)	107 (7.8)	95 (5.4)	104 (3.5)
43	Fipronil	83 (10)	100 (9.1)	107 (8.4)	104 (4.4)	106 (8.3)	113 (7.3)	80 (3.8)	92 (5.9)
44	Cyazofamid	116 (3.5)	98 (4.4)	105 (4.1)	102 (2.9)	105 (5.3)	106 (0.8)	94 (5.9)	103 (5.7)
45	Kresoxim-methyl	91 (13)	99 (3.0)	123 (9.2)	97 (11)	97 (13)	106 (2.2)	109 (15)	99 (13)
46	Triflumuron	85 (13)	96 (3.0)	128 (5.2)	98 (8.2)	82 (8.2)	78 (8.0)	109 (2.9)	93 (14)
47	Difenoconazole	104 (3.7)	98 (0.6)	93 (6.8)	98 (4.2)	94 (1.9)	92 (0.4)	101 (7.2)	100 (1.0)
48	Buprofezin	106 (5.6)	100 (3.3)	94 (12)	105 (7.7)	73 (7.2)	87 (2.6)	111 (3.6)	112 (0.3)
49	Diazinon	102 (4.4)	100 (1.2)	99 (2.1)	105 (1.3)	98 (4.0)	96 (1.1)	90 (1.5)	104 (1.5)
50	Pirimiphos-methyl	97 (4.0)	101 (2.2)	99 (4.1)	105 (1.2)	79 (9.0)	91 (3.0)	86 (6.9)	103 (1.8)
51	Isopyrazam	94 (8.6)	103 (1.3)	128 (13)	98 (2.5)	107 (2.6)	102 (2.5)	94 (9.2)	96 (6.9)
52	Profenofos	95 (6.4)	101 (0.5)	114 (8.5)	100 (1.6)	85 (4.0)	93 (3.4)	100 (4.3)	100 (2.1)
53	Indoxacarb	97 (8.7)	109 (1.5)	87 (13)	100 (4.5)	105 (12)	98 (0.8)	106 (1.4)	118 (4.6)
54	Trifloxystrobin	98 (2.2)	104 (1.4)	101 (6.8)	100 (1.9)	96 (6.4)	99 (0.9)	96 (3.9)	107 (0.8)
55	Pyriproxyfen	87 (2.9)	98 (0.9)	99 (0.8)	107 (1.0)	87 (2.2)	96 (1.9)	91 (0.7)	98 (0.9)
56	Chlorpyrifos	112 (13)	102 (3.9)	77 (12)	103 (1.1)	111 (6.2)	92 (4.0)	99 (13)	96 (1.4)
57	Hexythiazox	106 (3.2)	101 (2.6)	106 (2.3)	106 (1.2)	102 (0.2)	101 (1.0)	97 (1.4)	101 (2.1)
58	Pendimethalin	105 (4.3)	101 (2.3)	95 (5.4)	101 (1.8)	90 (5.4)	98 (0.5)	99 (5.2)	105 (2.7)
59	Fluroxypyr	105 (7.5)	97 (4.5)	120 (2.8)	105 (2.2)	82 (5.1)	88 (5.5)	93 (3.9)	98 (0.6)
60	Pyridaben	104 (6.7)	102 (1.4)	100 (5.1)	99 (4.4)	78 (0.9)	97 (0.4)	97 (3.3)	100 (0.9)

Table S2. MEs, LOQs and LODs of the studied pesticides in lettuce and Chinese chives.

No.	Pesticides	Lettuce						Chinese chives					
		Sin-QuEChERS Nano			d-SPE			Sin-QuEChERS Nano			d-SPE		
		ME	LOQ (μg/kg)	LOD (μg/kg)	ME	LOQ (μg/kg)	LOD (μg/kg)	ME	LOQ (μg/kg)	LOD (μg/kg)	ME	LOQ (μg/kg)	LOD (μg/kg)
**System: GC-MS/MS**													
1	Dichlorvos	2.05	1.8	0.5	2.68	2.7	0.8	3.41	2.2	0.7	3.50	2.3	0.7
2	Trichlorfon	2.04	2.4	0.7	2.66	0.9	0.3	3.41	1.7	0.5	3.56	1.9	0.6
3	Etridiazole	1.56	3.8	1.1	1.82	10	3.0	1.99	2.7	0.8	1.25	10.0	3.0
4	Carbaryl	1.50	0.6	0.2	1.40	0.7	0.2	1.24	3.1	0.9	1.27	1.7	0.5
5	Orthophenylphenol	1.50	2.8	0.8	1.34	2.8	0.8	1.98	10	3.0	1.80	10	3.0
6	Propachlor	1.48	1.4	0.4	1.83	1.3	0.4	1.57	1.3	0.4	1.73	1.2	0.4
7	Ethoprophos	1.50	3.6	1.1	1.59	4.3	1.3	1.61	4.5	1.4	1.70	4.0	1.2
8	Chlorpropham	1.41	4.3	1.3	1.45	4.4	1.3	1.71	4.4	1.3	1.61	4.3	1.3
9	Trifluralin	1.20	1.7	0.5	1.29	1.4	0.4	1.33	1.4	0.4	1.35	1.5	0.5
10	Sulfotep	1.28	5.2	1.6	1.28	5.0	1.5	1.25	4.5	1.4	1.27	5.2	1.6
11	Phorate	1.32	4.3	1.3	1.33	6.0	1.8	1.38	4.2	1.3	1.34	4.3	1.3
12	Atrazine	1.25	7.4	2.2	1.36	6.6	2.0	1.38	6.8	2.0	1.29	7.2	2.2
13	Propazine	1.14	4.3	1.3	1.19	3.2	1.0	1.20	3.9	1.2	1.13	3.7	1.1
14	Clomazone	1.27	3.0	0.9	1.30	2.6	0.8	1.30	2.1	0.6	1.18	2.8	0.8
15	Lindane	1.29	1.2	0.4	1.46	1.1	0.3	1.56	1.2	0.4	1.20	1.2	0.4
16	Propyzamide	1.30	1.5	0.5	1.32	1.4	0.4	1.29	6.7	2.0	1.29	5.6	1.7
17	Diazinon	1.19	3.9	1.2	1.22	3.7	1.1	1.22	1.8	0.5	1.14	3.6	1.1
18	Triallate	1.17	4.8	1.4	1.25	4.7	1.4	1.20	4.8	1.4	1.14	5.3	1.6
19	Pirimicarb	1.15	2.8	0.8	1.15	3.1	0.9	1.26	1.9	0.6	1.14	2.5	0.8
20	Propanil	1.32	1.3	0.4	1.25	1.4	0.4	1.34	5.2	1.6	1.40	4.5	1.4
21	Acetochlor	1.41	1.4	0.4	1.47	1.5	0.5	1.39	1.1	0.3	1.32	1.5	0.5

No.	Pesticides	Lettuce						Chinese chives					
		Sin-QuEChERS Nano			d-SPE			Sin-QuEChERS Nano			d-SPE		
		ME	LOQ (μg/kg)	LOD (μg/kg)	ME	LOQ (μg/kg)	LOD (μg/kg)	ME	LOQ (μg/kg)	LOD (μg/kg)	ME	LOQ (μg/kg)	LOD (μg/kg)
22	Propisochlor	1.36	1.1	0.3	1.50	1.0	0.3	1.37	1.4	0.4	1.28	1.3	0.4
23	Metribuzin	1.25	5.4	1.6	1.32	4.0	1.2	1.17	1.2	0.4	1.14	1.1	0.3
24	Vinclozolin	1.05	6.1	1.8	1.14	7.2	2.2	1.14	6.2	1.9	1.02	5.3	1.6
25	Parathion-Methyl	1.31	6.2	1.9	1.95	5.9	1.8	2.19	5.1	1.5	2.67	5.8	1.7
26	Tolclofos-Methyl	1.16	2.6	0.8	1.30	2.9	0.9	1.26	2.5	0.8	1.26	1.6	0.5
27	Metalaxyl	1.25	4.7	1.4	1.15	5.4	1.6	1.19	5.1	1.5	1.24	4.7	1.4
28	Ametryn	1.26	4.3	1.3	1.23	4.2	1.3	1.23	4.9	1.5	1.13	4.4	1.3
29	Prometryn	1.12	3.8	1.1	1.15	3.8	1.1	1.18	4.0	1.2	1.06	4.4	1.3
30	Pirimiphos-Methyl	1.19	1.2	0.4	1.35	1.1	0.3	1.29	5.7	1.7	1.23	5.6	1.7
31	Fenitrothion	1.34	2.0	0.6	1.72	2.5	0.8	1.99	8.5	2.6	2.25	9.0	2.7
32	Malathion	1.56	1.1	0.3	2.14	1.2	0.4	2.14	3.5	1.1	2.21	3.4	1.0
33	Metolachlor	1.19	1.6	0.5	1.26	1.6	0.5	1.18	1.0	0.3	1.14	1.0	0.3
34	Diethofencarb	1.34	3.5	1.1	1.54	3.9	1.2	1.56	3.8	1.1	1.54	3.2	1.0
35	Triadimefon	1.37	1.0	0.3	1.34	1.0	0.3	1.33	5.0	1.5	1.32	5.2	1.6
36	Thiametoxam	1.41	1.5	0.5	1.48	1.9	0.6	1.75	1.8	0.5	1.56	6.2	1.9
37	Pendimethalin	1.39	6.6	2.0	1.39	6.2	1.9	1.37	5.3	1.6	1.36	4.9	1.5
38	Fipronil	1.48	5.1	1.5	1.58	5.4	1.6	1.20	4.9	1.5	1.40	5.2	1.6
39	Penconazole	1.14	2.8	0.8	1.18	2.7	0.8	1.20	2.9	0.9	1.12	2.6	0.8
40	Chlorfenvinphos	1.23	5.4	1.6	1.69	4.8	1.4	1.63	1.1	0.3	2.05	4.0	1.2
41	Phenthoate	1.23	3.8	1.1	1.47	4.2	1.3	1.39	4.0	1.2	1.44	3.7	1.1
42	Triadimenol	1.24	3.2	1.0	1.35	3.7	1.1	1.31	6.8	2.0	1.32	7.8	2.3
43	Methidathion	1.55	1.5	0.5	2.44	1.4	0.4	2.42	6.5	2.0	3.24	6.2	1.9
44	Butachlor	1.32	0.8	0.2	1.42	1.3	0.4	1.26	4.9	1.5	1.34	5.2	1.6
45	Napropamide	1.19	4.1	1.2	1.27	4.1	1.2	1.31	5.1	1.5	1.26	4.4	1.3

No.	Pesticides	Lettuce						Chinese chives					
		Sin-QuEChERS Nano			d-SPE			Sin-QuEChERS Nano			d-SPE		
		ME	LOQ (μg/kg)	LOD (μg/kg)	ME	LOQ (μg/kg)	LOD (μg/kg)	ME	LOQ (μg/kg)	LOD (μg/kg)	ME	LOQ (μg/kg)	LOD (μg/kg)
46	Pretilachlor	1.25	3.1	0.9	1.44	3.2	1.0	1.23	5.3	1.6	1.28	2.1	0.6
47	Isoprothiolane	1.12	4.5	1.4	1.18	4.2	1.3	1.19	5.9	1.8	1.18	4.3	1.3
48	Oxadiazon	1.08	4.4	1.3	1.15	4.0	1.2	1.09	4.1	1.2	1.01	3.8	1.1
49	Thifluzamide	1.72	3.3	1.0	1.94	2.5	0.8	1.90	3.3	1.0	1.90	2.6	0.8
50	Myclobutanil	1.26	2.6	0.8	1.29	2.4	0.7	1.26	2.4	0.7	1.28	2.2	0.7
51	o,p'-DDT	1.34	1.9	0.6	1.24	2.2	0.7	1.03	3.0	0.9	1.04	2.5	0.8
52	Kresoxim-Methyl	1.23	4.4	1.3	1.24	4.4	1.3	1.26	4.9	1.5	1.15	4.3	1.3
53	Phosmet	1.15	2.5	0.8	1.20	2.2	0.7	1.14	2.1	0.6	1.17	2.6	0.8
54	Trifloxystrobin	1.16	2.6	0.8	1.22	2.7	0.8	1.18	2.2	0.7	1.16	1.5	0.5
55	Cyproconazole	1.41	4.2	1.3	1.51	4.5	1.4	1.44	4.3	1.3	1.50	3.5	1.1
56	Diniconazole	1.40	4.1	1.2	1.50	3.7	1.1	1.46	4.9	1.5	1.50	4.4	1.3
57	Oxadixyl	1.15	3.5	1.1	1.38	3.2	1.0	1.39	3.2	1.0	1.41	3.0	0.9
58	Carfentrazone-ethyl	1.42	2.0	0.6	1.55	2.2	0.7	1.61	2.0	0.6	1.55	2.1	0.6
59	Clodinafop-propargyl	1.04	4.8	1.4	1.35	4.5	1.4	1.43	4.5	1.4	1.43	4.4	1.3
60	Tebuconazole	1.26	3.3	1.0	1.40	3.1	0.9	1.31	3.4	1.0	1.39	3.3	1.0
61	Diclofopmethyl	1.07	3.4	1.0	1.16	3.7	1.1	1.12	4.0	1.2	1.10	3.7	1.1
62	Propargite	1.41	1.2	0.4	1.77	1.4	0.4	1.70	1.5	0.5	1.82	1.7	0.5
63	Bifenthrin	1.28	0.9	0.3	1.34	0.8	0.2	1.30	0.9	0.3	1.29	0.8	0.2
64	Cypermethrin	1.34	1.6	0.5	1.53	1.6	0.5	1.56	7.7	2.3	1.60	7.9	2.4
65	Fenpropathrin	1.24	6.2	1.9	1.48	4.9	1.5	1.42	5.8	1.7	1.41	5.8	1.7
66	Lambda-Cyhalothrin	1.47	5.7	1.7	1.64	5.0	1.5	1.87	6.0	1.8	1.57	5.8	1.7
67	Triticonazole	1.59	6.0	1.8	1.91	5.6	1.7	1.77	5.3	1.6	2.17	4.3	1.3
68	Pyriproxyfen	1.32	3.2	1.0	1.42	3.8	1.1	1.41	3.9	1.2	1.43	4.5	1.4
69	Cyhalofopbutyl	1.19	2.6	0.8	1.42	2.4	0.7	1.39	2.5	0.8	1.39	2.6	0.8

No.	Pesticides	Lettuce						Chinese chives					
		Sin-QuEChERS Nano			d-SPE			Sin-QuEChERS Nano			d-SPE		
		ME	LOQ (μg/kg)	LOD (μg/kg)	ME	LOQ (μg/kg)	LOD (μg/kg)	ME	LOQ (μg/kg)	LOD (μg/kg)	ME	LOQ (μg/kg)	LOD (μg/kg)
70	Permethrin	1.39	1.6	0.5	1.58	6.4	1.9	1.53	1.4	0.4	1.56	3.1	0.9
71	Pyridaben	1.45	2.8	0.8	1.76	2.5	0.8	1.57	1.4	0.4	1.72	1.6	0.5
72	Beta-cypermethrin	1.53	1.7	0.5	1.91	1.7	0.5	2.74	6.8	2.0	2.00	5.2	1.6
73	Flumioxazin	1.71	1.4	0.4	2.56	1.6	0.5	2.28	6.2	1.9	3.23	5.6	1.7
74	Esfenvalerate	1.79	1.3	0.4	2.49	1.3	0.4	2.79	7.0	2.1	2.92	3.7	1.1
75	Famoxadone	1.25	1.6	0.5	2.12	1.9	0.6	1.96	8.6	2.6	2.91	5.4	1.6
**System: LC-MS/MS**													
1	Omethoate	1.05	2.7	0.8	1.24	2.6	0.8	0.98	3.1	0.9	0.88	3.2	1.0
2	Methomyl	1.01	3.9	1.2	0.89	5.0	1.5	0.88	8.6	2.6	0.76	10	3.0
3	Thiamethoxam	0.95	10	3.0	0.94	10	3.0	0.96	4.4	1.3	0.87	4.9	1.5
4	Metamitron	0.96	2.4	0.7	0.96	2.2	0.7	0.87	2.9	0.9	0.75	2.5	0.8
5	Clothianidin	1.01	8.4	2.5	0.98	4.5	1.4	0.98	3.9	1.2	0.89	4.5	1.4
6	Imidacloprid	1.07	3.7	1.1	1.02	2.7	0.8	1.01	2.6	0.8	0.95	3.4	1.0
7	Dimethoate	0.98	2.1	0.6	0.95	1.0	0.3	0.94	1.8	0.5	0.85	1.8	0.5
8	Acetamiprid	0.95	3.8	1.1	0.93	1.9	0.6	0.89	1.9	0.6	0.80	2.0	0.6
9	Cymoxanil	1.04	7.4	2.2	0.93	3.1	0.9	0.75	4.3	1.3	0.63	5.3	1.6
10	Thiacloprid	1.00	3.2	1.0	0.96	1.6	0.5	0.95	2.3	0.7	0.83	1.8	0.5
11	Imazalil	0.90	1.0	0.3	0.80	1.1	0.3	0.72	1.4	0.4	0.65	1.6	0.5
12	Metribuzin	0.98	1.2	0.4	0.97	1.7	0.5	0.87	1.5	0.5	0.84	2.4	0.7
13	Bendiocarb	0.98	2.7	0.8	0.91	7.7	2.3	0.87	3.6	1.1	0.81	3.6	1.1
14	Carbofuran	1.06	1.1	0.3	1.04	1.0	0.3	0.99	1.0	0.3	0.93	2.0	0.6
15	Carbaryl	0.96	1.8	0.5	0.89	1.6	0.5	0.92	1.9	0.6	0.85	1.9	0.6
16	Isoproturon	0.97	1.4	0.4	0.90	1.7	0.5	0.85	1.9	0.6	0.79	1.4	0.4
17	Metalaxyl	1.18	1.8	0.5	1.10	2.2	0.7	1.09	1.7	0.5	1.14	2.0	0.6

No.	Pesticides	Lettuce						Chinese chives					
		Sin-QuEChERS Nano			d-SPE			Sin-QuEChERS Nano			d-SPE		
		ME	LOQ (μg/kg)	LOD (μg/kg)	ME	LOQ (μg/kg)	LOD (μg/kg)	ME	LOQ (μg/kg)	LOD (μg/kg)	ME	LOQ (μg/kg)	LOD (μg/kg)
18	Propanil	1.03	2.1	0.6	0.98	1.9	0.6	0.93	2.2	0.7	0.87	2.2	0.7
19	Dimethomorph	1.07	4.1	1.2	0.99	4.0	1.2	0.98	3.9	1.2	0.98	3.5	1.1
20	Cyprodinil	0.96	1.3	0.4	0.92	1.0	0.3	0.80	2.0	0.6	0.79	1.2	0.4
21	Triadimenol	1.02	1.9	0.6	0.97	1.8	0.5	0.95	2.2	0.7	0.92	2.2	0.7
22	Cyproconazole	1.02	1.4	0.4	0.98	1.5	0.5	0.97	1.6	0.5	0.92	1.7	0.5
23	Fludioxonil	1.09	2.8	0.8	0.98	2.6	0.8	0.98	2.2	0.7	0.91	1.8	0.5
24	Triticonazole	1.08	1.4	0.4	1.05	1.3	0.4	1.03	1.5	0.5	1.08	1.7	0.5
25	Diethofencarb	1.08	2.0	0.6	0.99	2.0	0.6	0.98	2.8	0.8	0.92	3.0	0.9
26	Spirotetramat	1.06	1.7	0.5	1.01	2.0	0.6	0.97	2.1	0.6	1.07	4.8	1.4
27	Prochloraz	1.07	1.8	0.5	0.95	1.6	0.5	1.00	1.9	0.6	0.95	1.4	0.4
28	Myclobutanil	1.06	0.3	0.1	1.05	1.3	0.4	1.03	0.9	0.3	1.05	1.1	0.3
29	Azoxystrobin	1.11	0.9	0.3	1.08	1.1	0.3	1.07	0.8	0.2	1.09	0.9	0.3
30	Triadimefon	1.09	1.9	0.6	1.04	2.4	0.7	1.05	5.0	1.5	1.05	5.0	1.5
31	Epoxiconazole	1.07	1.2	0.4	1.04	1.1	0.3	1.01	1.3	0.4	0.96	1.1	0.3
32	Ethoprophos	1.06	3.0	0.9	1.01	2.9	0.9	0.96	2.7	0.8	0.95	2.8	0.8
33	Fluopyram	1.06	1.3	0.4	1.04	1.8	0.5	0.99	1.4	0.4	0.97	1.4	0.4
34	Tebuconazole	1.05	1.2	0.4	1.05	1.1	0.3	1.01	1.2	0.4	1.02	1.3	0.4
35	Hexaconazole	1.11	2.3	0.7	1.07	1.3	0.4	1.05	1.4	0.4	1.04	1.5	0.5
36	Penconazole	1.00	1.5	0.5	0.98	3.1	0.9	0.94	1.2	0.4	0.87	3.5	1.1
37	Triazophos	1.00	1.7	0.5	1.00	0.9	0.3	0.97	1.5	0.5	0.93	1.4	0.4
38	Metolachlor	1.06	0.7	0.2	1.03	0.4	0.1	1.01	0.6	0.2	0.96	0.6	0.2
39	Diniconazole	1.09	1.3	0.4	1.06	1.2	0.4	1.03	1.6	0.5	1.03	1.5	0.5
40	Propiconazole	1.05	1.2	0.4	1.03	1.2	0.4	0.99	1.3	0.4	0.93	1.4	0.4
41	Tebufenozide	1.06	1.7	0.5	1.05	1.5	0.5	1.10	1.9	0.6	1.04	2.1	0.6

No.	Pesticides	Lettuce						Chinese chives					
		Sin-QuEChERS Nano			d-SPE			Sin-QuEChERS Nano			d-SPE		
		ME	LOQ (μg/kg)	LOD (μg/kg)	ME	LOQ (μg/kg)	LOD (μg/kg)	ME	LOQ (μg/kg)	LOD (μg/kg)	ME	LOQ (μg/kg)	LOD (μg/kg)
42	Chlorfenvinphos	1.00	3.0	0.9	1.02	2.4	0.7	0.99	2.3	0.7	0.90	2.2	0.7
43	Fipronil	0.92	1.8	0.5	0.82	1.7	0.5	0.94	1.7	0.5	0.99	1.8	0.5
44	Cyazofamid	1.01	2.5	0.8	0.96	3.4	1.0	0.94	3.5	1.1	0.94	3.8	1.1
45	Kresoxim-methyl	1.02	3.8	1.1	1.01	2.3	0.7	0.92	2.9	0.9	0.96	2.6	0.8
46	Triflumuron	0.99	4.4	1.3	0.83	1.3	0.4	0.93	4.2	1.3	0.83	3.1	0.9
47	Difenoconazole	1.12	1.2	0.4	1.08	1.2	0.4	1.06	1.2	0.4	1.03	1.2	0.4
48	Buprofezin	1.01	1.9	0.6	0.92	1.8	0.5	0.89	3.4	1.0	0.71	2.7	0.8
49	Diazinon	1.04	0.5	0.2	1.01	0.5	0.2	0.97	0.4	0.1	0.92	0.4	0.1
50	Pirimiphos-methyl	1.01	0.8	0.2	0.94	0.8	0.2	0.97	1.3	0.4	0.82	1.3	0.4
51	Isopyrazam	1.07	1.6	0.5	1.05	1.9	0.6	1.03	1.9	0.6	1.04	1.9	0.6
52	Profenofos	0.98	1.1	0.3	0.98	1.0	0.3	0.92	1.1	0.3	0.85	1.1	0.3
53	Indoxacarb	1.11	1.6	0.5	1.06	1.6	0.5	1.04	2.0	0.6	0.97	2.0	0.6
54	Trifloxystrobin	1.06	1.8	0.5	1.05	1.6	0.5	1.00	1.7	0.5	0.97	5.0	1.5
55	Pyriproxyfen	1.03	0.7	0.2	0.99	0.6	0.2	0.97	0.7	0.2	0.91	0.7	0.2
56	Chlorpyrifos	1.02	2.8	0.8	0.92	2.9	0.9	0.98	10	3.0	0.92	5.0	1.5
57	Hexythiazox	1.07	1.0	0.3	1.03	0.9	0.3	1.03	1.3	0.4	0.98	2.5	0.8
58	Pendimethalin	1.17	0.9	0.3	1.09	0.9	0.3	1.11	0.9	0.3	1.09	1.0	0.3
59	Fluroxypyr	1.07	2.4	0.7	1.04	2.7	0.8	0.97	5.0	1.5	0.91	3.9	1.2
60	Pyridaben	1.27	0.6	0.2	1.18	0.7	0.2	1.02	0.7	0.2	1.00	0.6	0.2

Table S3. GC-MS/MS quantification and qualitative transitions, collision energy and retention times (Rts) used for studied pesticides.

No.	Pesticide	Rt (min)	Quantification transition*	Qualitative transition*
1	Dichlorvos	4.55	109→79 (10)	185→93 (10)
2	Trichlorfon	4.54	109→79 (5)	145→109 (10)
3	Etridiazole	5.54	211→140 (20)	213→185 (10)
4	Carbaryl	5.89	144→115 (20)	144→116 (20)
5	Orthophenylphenol	5.90	170→169 (10)	170→141 (20)
6	Propachlor	6.34	176→120 (10)	196→120 (10)
7	Ethoprophos	6.48	158→97 (15)	200→158 (5)
8	Chlorpropham	6.61	213→127 (15)	213→171 (8)
9	Trifluralin	6.61	306→264 (5)	264→160 (15)
10	Sulfotep	6.67	238→146 (13)	322→202 (12)
11	Phorate	6.83	121→65 (10)	260→75 (5)
12	Atrazine	7.17	215→200 (10)	200→104 (20)
13	Propazine	7.21	214→172 (10)	229→214 (10)
14	Clomazone	7.22	125→89 (15)	204→107 (15)
15	Lindane	7.33	181→145 (15)	183→147 (15)
16	Propyzamide	7.40	240→173 (12)	255→240 (6)
17	Diazinon	7.40	137→84 (10)	179→137 (15)
18	Triallate	7.67	268→184 (20)	270→186 (15)
19	Pirimicarb	7.74	238→166 (10)	238→72 (28)
20	Propanil	8.04	161→99 (25)	217→161 (10)
21	Acetochlor	8.05	223→146 (10)	146→117 (15)
22	Propisochlor	8.05	223→147 (10)	162→144 (10)
23	Metribuzin	8.07	198→82 (15)	198→89 (10)
24	Vinclozolin	8.12	285→212 (12)	285→178 (13)
25	Parathion-Methyl	8.16	263→109 (15)	233→124 (15)
26	Tolclofos-Methyl	8.18	265→250 (10)	265→220 (22)
27	Metalaxyl	8.27	234→174 (10)	249→190 (10)
28	Ametryn	8.27	227→170 (10)	227→212 (10)
29	Prometryn	8.31	241→184 (13)	241→199 (8)
30	Pirimiphos-Methyl	8.47	290→125 (15)	290→233 (10)
31	Fenitrothion	8.52	277→109 (20)	277→260 (10)
32	Malathion	8.63	127→99 (10)	173→99 (10)
33	Metolachlor	8.73	162→133 (15)	238→162 (15)
34	Diethofencarb	8.77	267→225 (8)	267→168 (18)
35	Triadimefon	8.92	208→181 (10)	208→127 (10)
36	Thiametoxam	9.19	212→139 (15)	247→212 (10)
37	Pendimethalin	9.27	252→162 (12)	252→191 (12)
38	Fipronil	9.35	367→213 (16)	213→178 (10)
39	Penconazole	9.36	248→157 (25)	248→192 (13)
40	Chlorfenvinphos	9.42	267→159 (15)	323→267 (15)
41	Phenthoate	9.50	274→121 (7)	246→121 (10)
42	Triadimenol	9.59	168→70 (10)	128→100 (10)

No.	Pesticide	Rt (min)	Quantification transition*	Qualitative transition*
43	Methidathion	9.75	145→85 (10)	145→58 (15)
44	Butachlor	9.91	237→160 (10)	176→146 (10)
45	Napropamide	10.13	271→72 (15)	128→100 (8)
46	Pretilachlor	10.24	162→132 (15)	162→147 (15)
47	Isoprothiolane	10.23	290→118 (15)	290→204 (15)
48	Oxadiazon	10.36	258→175 (10)	304→260 (10)
49	Thifluzamide	10.39	194→166 (12)	449→429 (10)
50	Myclobutanil	10.42	179→125 (15)	179→152 (15)
51	o,p'-DDT	10.48	235→165 (22)	235→199 (13)
52	Kresoxim-Methyl	10.49	206→131 (15)	206→89 (30)
53	Phosmet	10.49	116→89 (10)	116→88 (23)
54	Trifloxystrobin	10.49	116→89 (15)	131→130 (10)
55	Cyproconazole	10.72	222→125 (20)	224→127 (20)
56	Diniconazole	10.99	268→232 (15)	270→234 (15)
57	Oxadixyl	11.02	163→132 (14)	233→146 (20)
58	Carfentrazone-ethyl	11.46	330→310 (20)	411→340 (10)
59	Clodinafop-propargyl	11.71	349→238 (15)	349→266 (15)
60	Tebuconazole	11.94	250→125 (20)	252→127 (20)
61	Diclofopmethyl	11.97	253→162 (15)	340→253 (15)
62	Propargite	11.97	135→107 (15)	173→135 (12)
63	Bifenthrin	12.55	181→166 (15)	181→141 (22)
64	Cypermethrin	12.71	181→152 (22)	209→116 (13)
65	Fenpropathrin	12.72	265→210 (15)	181→152 (23)
66	Lambda-Cyhalothrin	12.71, 13.50	208→181 (10)	181→152 (23)
67	Triticonazole	13.13	235→182 (10)	235→217 (10)
68	Pyriproxyfen	13.31	136→78 (15)	136→96 (15)
69	Cyhalofopbutyl	13.36	256→120 (10)	357→256 (10)
70	Permethrin	14.25, 14.38	183→153 (15)	183→168 (15)
71	Pyridaben	14.39	147→117 (22)	147→132 (13)
72	Beta-cypermethrin	15.26, 15.31	181→152 (25)	163→127 (10)
73	Flumioxazin	15.89	287→259 (15)	354→326 (10)
74	Esfenvalerate	15.93, 16.12	167→125 (10)	167→139 (10)
75	Famoxadone	17.04	330→224 (10)	224→196 (10)

* Collision energy (eV) is given in brackets.

Table S4. LC-MS/MS quantification and qualitative transitions, collision energy, tube lens voltages and retention times (Rts) used for studied pesticides.

No.	Pesticide	Rt (min)	ESI	Tube lens voltage (V)	Quantification transition*	Qualitative transition*	
1	Omethoate	0.82	+	75	214.0→125.1 (20)	214.0→155.1 (14)	214.0→182.1 (9)
2	Methomyl	1.96	+	50	163.1→106.2 (9)	163.1→88.2 (7)	
3	Thiamethoxam	2.33	+	78	292.0→211.1 (11)	292.0→181.1 (21)	
4	Metamitron	2.88	+	89	203.1→175.1 (16)	203.1→104.2 (21)	
5	Clothianidin	2.96	+	88	250.0→169.1 (13)	250.0→132.1 (17)	
6	Imidacloprid	3.29	+	71	256.0→209.1 (16)	256.0→175.1 (17)	
7	Dimethoate	3.50	+	57	230.0→125.1 (22)	230.0→199.0 (7)	
8	Acetamiprid	3.69	+	83	223.1→126.1 (21)	223.1→90.2 (35)	
9	Cymoxanil	4.22	+	63	199.1→128.1 (7)	199.1→83.1 (23)	199.1→111.1 (17)
10	Thiacloprid	4.70	+	71	253.0→126.1 (21)	253.0→99.2 (45)	
11	Imazalil	5.38	+	114	297.2→159.1 (21)	297.2→69.2 (20)	297.2→201.1 (16)
12	Metribuzin	5.84	+	86	215.1→187.3 (16)	215.1→60.2 (53)	215.1→131.2 (19)
13	Bendiocarb	6.39	+	49	224.1→109.2 (18)	224.1→167.1 (6)	
14	Carbofuran	6.46	+	62	222.1→123.2 (21)	222.1→165.1 (11)	
15	Carbaryl	6.98	+	49	202.1→145.1 (10)	202.1→127.2 (28)	
16	Isoproturon	7.38	+	65	207.2→72.3 (18)	207.2→165.1 (14)	
17	Metalaxyl	7.44	+	86	280.1→220.1 (13)	280.1→160.1 (23)	280.1→192.1 (17)
18	Propanil	8.66	+	62	218.0→162.0 (15)	218.0→127.1 (25)	
19	Dimethomorph	8.70	+	94	388.1→301.1 (20)	388.1→165.1 (31)	
20	Cyprodinil	8.73	+	103	226.1→93.1 (33)	226.1→77.1 (41)	
21	Triadimenol	8.81	+	74	296.0→70.1 (20)	296.0→99.1 (15)	
22	Cyproconazole	9.21	+	93	292.0→70.1 (19)	292.0→89.1 (55)	292.0→125.1 (25)
23	Fludioxonil	9.36	-	75	247.0→180.1 (34)	247.0→126.1 (38)	247.0→169.1 (41)
24	Triticonazole	9.27	+	76	318.1→70.3 (17)	318.1→125.1 (27)	
25	Diethofencarb	9.38	+	53	268.1→180.1 (18)	268.1→226.1 (7)	

No.	Pesticide	Rt (min)	ESI	Tube lens voltage (V)	Quantification transition*	Qualitative transition*	
26	Spirotetramat	9.46	+	112	374.2→216.3 (28)	374.2→270.3 (19)	374.2→302.4 (13)
27	Prochloraz	9.47	+	95	378.1→310.1 (10)	378.1→70.1 (25)	378.1→268.1 (14)
28	Myclobutanil	9.74	+	98	289.1→70.1 (18)	289.1→125.0 (32)	
29	Azoxystrobin	9.92	+	78	404.1→372.2 (14)	404.1→344.2 (24)	
30	Triadimefon	9.98	+	85	294.1→69.1 (20)	294.1→225.1 (12)	294.1→197.1 (14)
31	Epoxiconazole	10.04	+	93	330.1→121.1 (20)	330.1→101.2 (40)	
32	Ethoprophos	10.06	+	67	243.1→131.0 (20)	243.1→173.0 (14)	
33	Fluopyram	10.44	+	115	397.1→208.2 (21)	397.1→145.2 (47)	
34	Tebuconazole	10.38	+	129	307.9→70.1 (18)	307.9→151.0 (25)	
35	Hexaconazole	10.67	+	75	314.0→70.3 (20)	314.0→159.0 (31)	
36	Penconazole	10.74	+	74	284.1→70.3 (17)	284.1→159.0 (31)	
37	Triazophos	10.87	+	84	314.0→162.1 (17)	314.0→97.0 (10)	314.0→119.1 (31)
38	Metolachlor	10.91	+	65	284.1→252.1 (15)	284.1→176.2 (25)	
39	Diniconazole	11.13	+	76	326.0→70.3 (24)	326.0→159.0 (32)	
40	Propiconazole	11.20	+	112	342.1→159.0 (28)	342.1→205.0 (20)	
41	Tebufenozide	11.52	+	102	353.2→133.2 (18)	353.2→105.2 (34)	
42	Chlorfenvinphos	11.53	+	63	361.0→99.1 (29)	361.0→155.1 (12)	
43	Fipronil	11.68	-	65	435.0→329.9 (18)	435.0→249.9 (29)	
44	Cyazofamid	11.72	+	57	325.1→108.2 (15)	325.1→261.1 (7)	
45	Kresoxim-methyl	11.80	+	80	314.1→222.1 (14)	314.1→267.1 (5)	314.1→235.1 (16)
46	Triflumuron	11.92	-	61	357.1→154.1 (15)	357.1→176.0 (24)	
47	Difenoconazole	12.01	+	89	406.1→251.0 (25)	406.1→188.1 (43)	406.1→337.0 (15)
48	Buprofezin	12.18	+	79	306.2→201.1 (11)	306.2→116.2 (16)	
49	Diazinon	12.40	+	82	305.0→169.1 (18)	305.0→153.2 (17)	305.0→97.0 (32)
50	Pirimiphos-methyl	12.48	+	89	306.0→67.1 (39)	306.0→164.2 (20)	306.0→108.1 (27)
51	Isopyrazam	12.73	+	85	360.1→244.2 (21)	360.1→258.2 (18)	360.1→320.3 (14)
52	Profenofos	13.31	+	90	372.9→302.9 (19)	372.9→345.0 (12)	

No.	Pesticide	Rt (min)	ESI	Tube lens voltage (V)	Quantification transition*	Qualitative transition*	
53	Indoxacarb	13.25	+	110	528.1→202.9 (37)	528.1→249.0 (15)	528.1→293.0 (13)
54	Trifloxystrobin	13.40	+	89	409.1→145.1 (45)	409.1→206.2 (12)	
55	Pyriproxyfen	14.43	+	90	322.0→96.1 (17)	322.0→185.1 (20)	322.0→78.1 (48)
56	Chlorpyrifos	14.70	+	89	349.8→198.0 (18)	349.8→107.0 (54)	349.8→97.0 (28)
57	Hexythiazox	14.77	+	100	353.2→168.2 (24)	353.2→115.2 (56)	
58	Pendimethalin	14.73	+	66	282.1→212.1 (9)	282.1→194.1 (16)	282.1→91.1 (24)
59	Fluroxypyr	15.29	+	96	254.9→209.0 (14)	254.9→181.0 (20)	254.9→179.1 (27)
60	Pyridaben	16.10	+	69	365.1→147.2 (24)	365.1→309.1 (12)	

* Collision energy (eV) is given in brackets.
